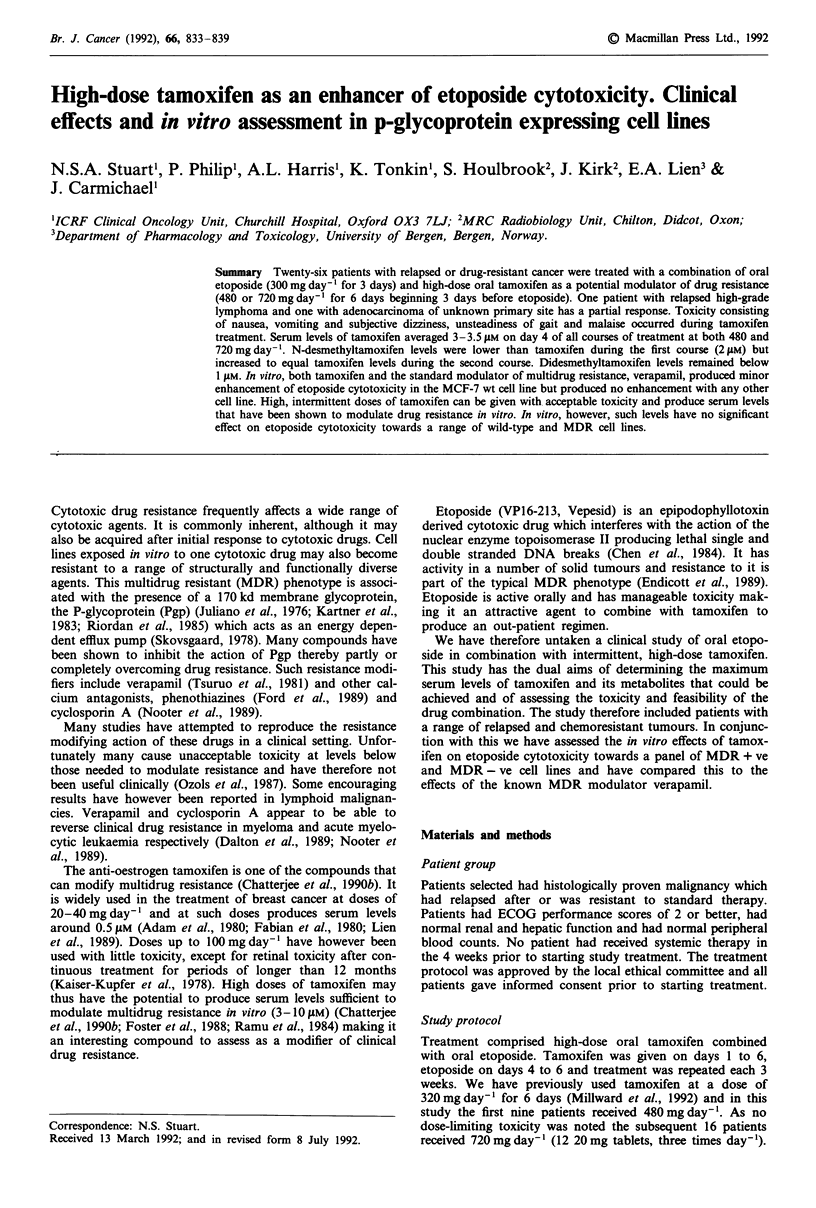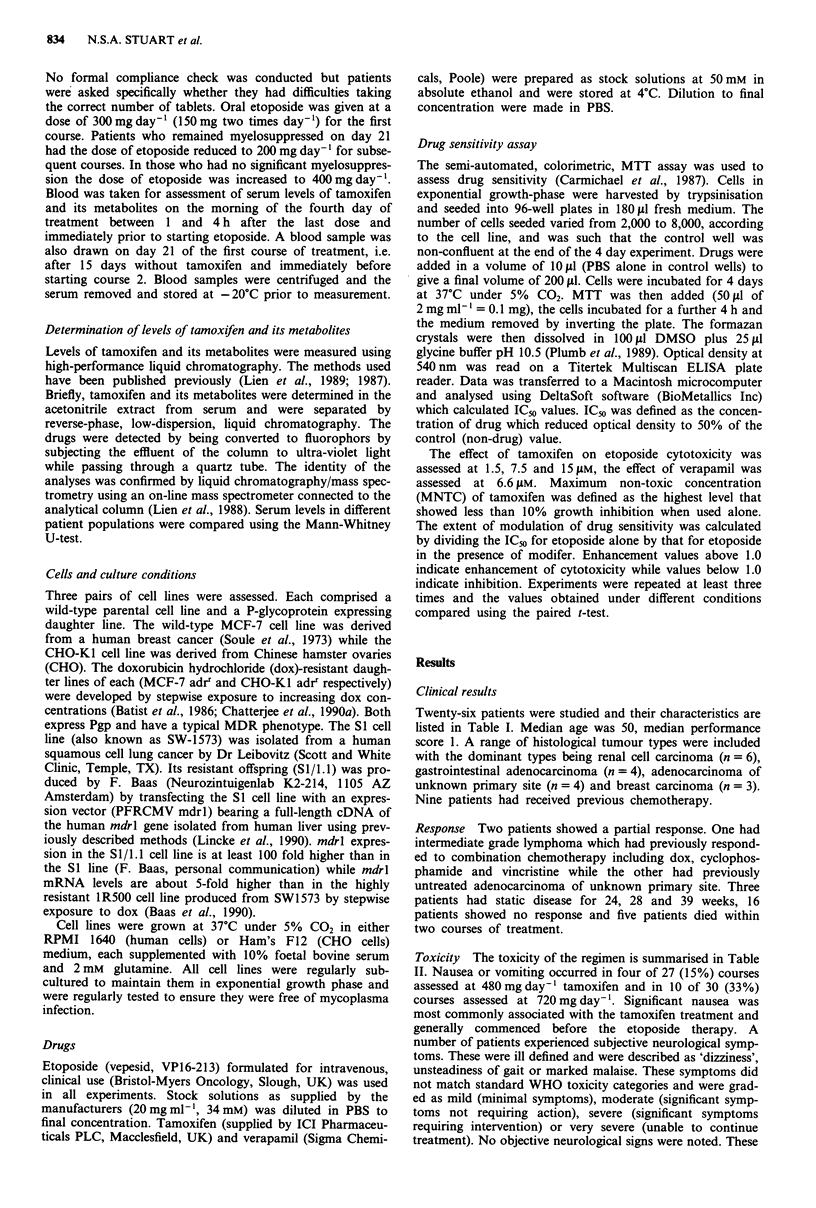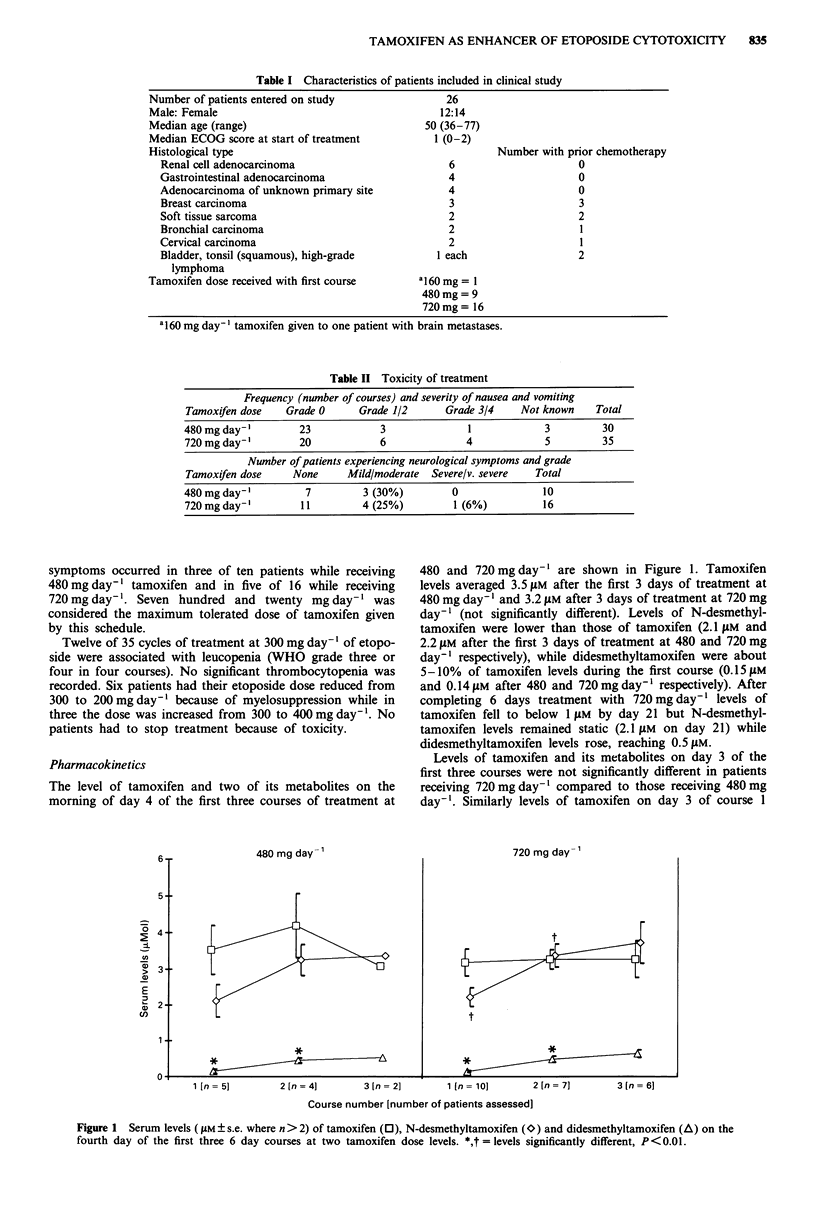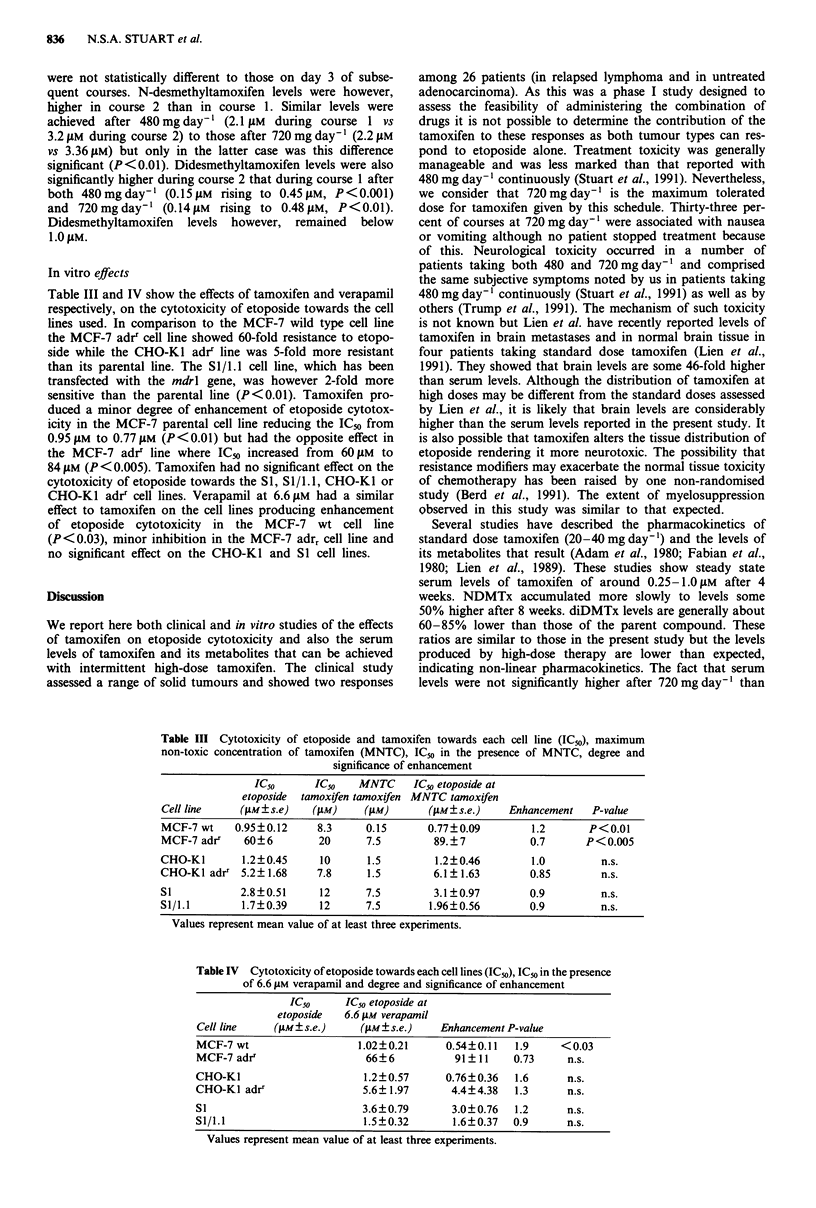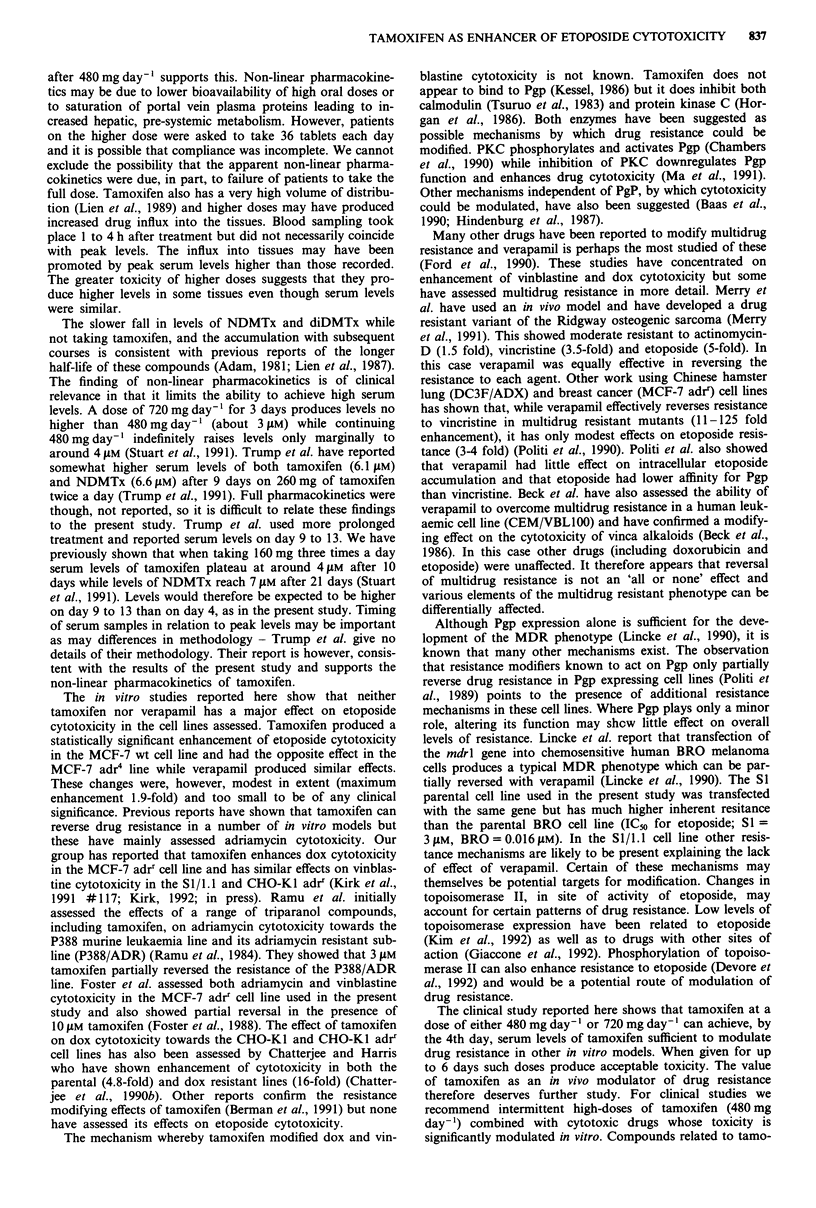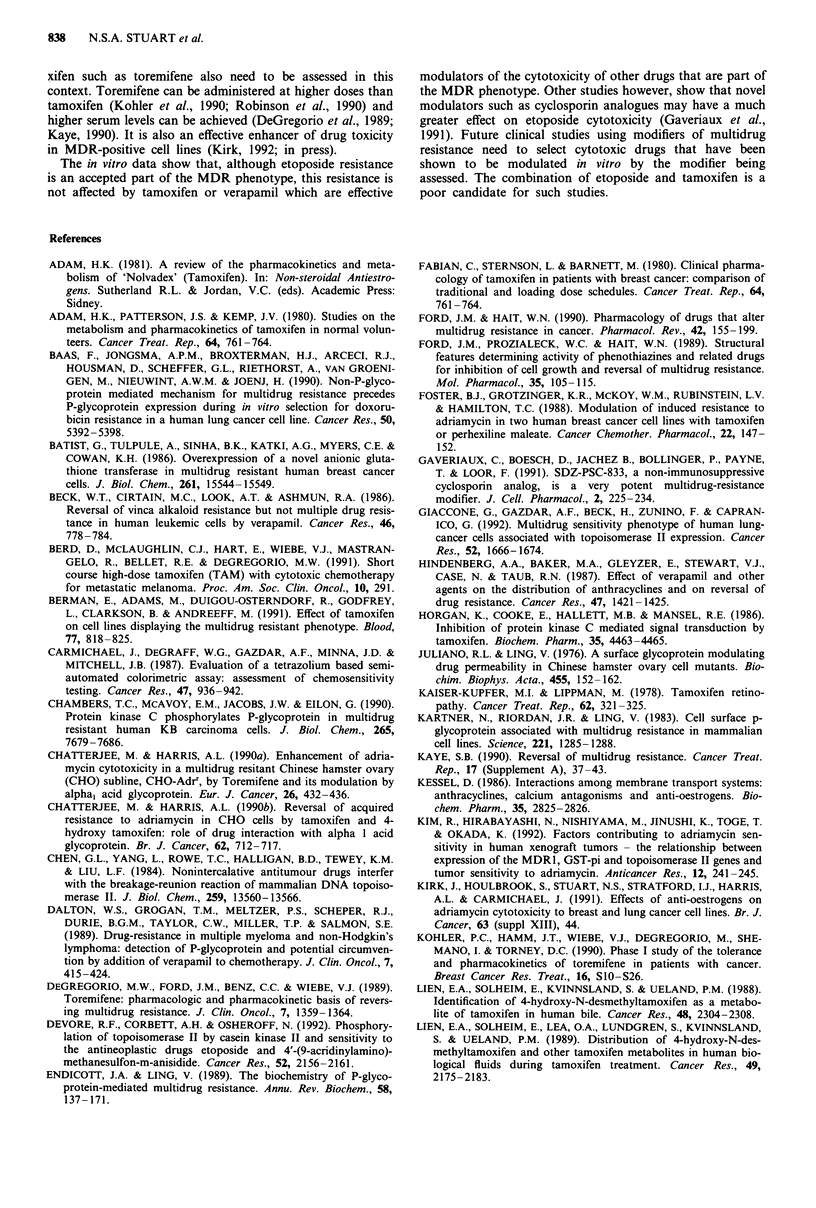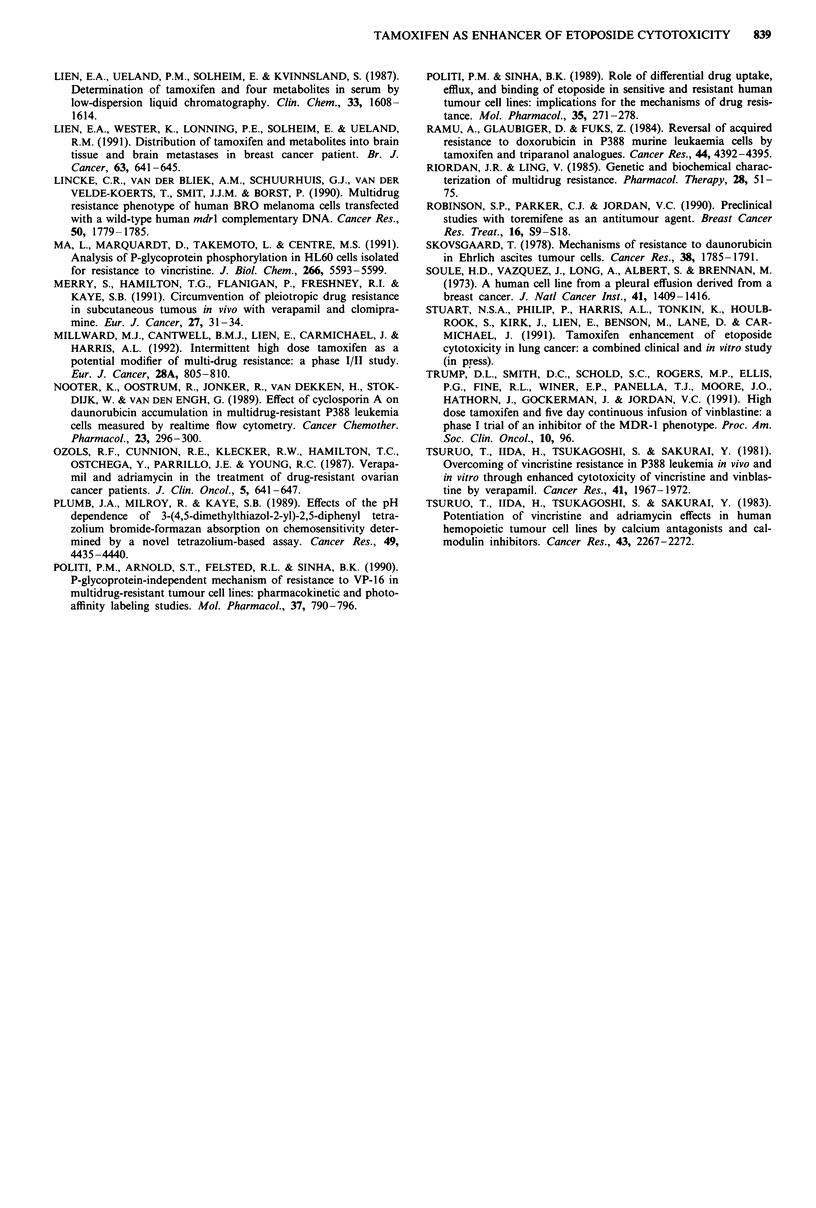# High-dose tamoxifen as an enhancer of etoposide cytotoxicity. Clinical effects and in vitro assessment in p-glycoprotein expressing cell lines.

**DOI:** 10.1038/bjc.1992.369

**Published:** 1992-11

**Authors:** N. S. Stuart, P. Philip, A. L. Harris, K. Tonkin, S. Houlbrook, J. Kirk, E. A. Lien, J. Carmichael

**Affiliations:** ICRF Clinical Oncology Unit, Churchill Hospital, Oxford.

## Abstract

Twenty-six patients with relapsed or drug-resistant cancer were treated with a combination of oral etoposide (300 mg day-1 for 3 days) and high-dose oral tamoxifen as a potential modulator of drug resistance (480 or 720 mg day-1 for 6 days beginning 3 days before etoposide). One patient with relapsed high-grade lymphoma and one with adenocarcinoma of unknown primary site has a partial response. Toxicity consisting of nausea, vomiting and subjective dizziness, unsteadiness of gait and malaise occurred during tamoxifen treatment. Serum levels of tamoxifen averaged 3-3.5 microM on day 4 of all courses of treatment at both 480 and 720 mg day-1. N-desmethyltamoxifen levels were lower than tamoxifen during the first course (2 microM) but increased to equal tamoxifen levels during the second course. Didesmethyltamoxifen levels remained below 1 microM. In vitro, both tamoxifen and the standard modulator of multidrug resistance, verapamil, produced minor enhancement of etoposide cytotoxicity in the MCF-7 wt cell line but produced no enhancement with any other cell line. High, intermittent doses of tamoxifen can be given with acceptable toxicity and produce serum levels that have been shown to modulate drug resistance in vitro. In vitro, however, such levels have no significant effect on etoposide cytotoxicity towards a range of wild-type and MDR cell lines.


 © Macmillan Press Ltd., 1992

### High-dose tamoxifen as an enhancer of etoposide cytotoxicity. Clinical effects and *in vitro* assessment in p-glycoprotein expressing cell lines

N.S.A. Stuart[1], P. Philip[1], A.L. Harris[1], K. Tonkin[1], S. Houlbrook[2], J. Kirk[2], E.A. Lien[3] & J. Carmichael[1]

[1]*ICRF Clinical Oncology Unit, Churchill Hospital, Oxford OX3 7LJ;* [2]*MRC Radiobiology Unit, Chilton, Didcot, Oxon;* [3]*Department of Pharmacology and Toxicology, University of Bergen, Bergen, Norway.*

**Summary** Twenty-six patients with relapsed or drug-resistant cancer were treated with a combination of oral etoposide (300 mg day$^{-1}$ for 3 days) and high-dose oral tamoxifen as a potential modulator of drug resistance (480 or 720 mg day$^{-1}$ for 6 days beginning 3 days before etoposide). One patient with relapsed high-grade lymphoma and one with adenocarcinoma of unknown primary site has a partial response. Toxicity consisting of nausea, vomiting and subjective dizziness, unsteadiness of gait and malaise occurred during tamoxifen treatment. Serum levels of tamoxifen averaged 3-3.5 μM on day 4 of all courses of treatment at both 480 and 720 mg day$^{-1}$. N-desmethyltamoxifen levels were lower than tamoxifen during the first course (2 μM) but increased to equal tamoxifen levels during the second course. Didesmethyltamoxifen levels remained below 1 μM. *In vitro*, both tamoxifen and the standard modulator of multidrug resistance, verapamil, produced minor enhancement of etoposide cytotoxicity in the MCF-7 wt cell line but produced no enhancement with any other cell line. High, intermittent doses of tamoxifen can be given with acceptable toxicity and produce serum levels that have been shown to modulate drug resistance *in vitro*. *In vitro*, however, such levels have no significant effect on etoposide cytotoxicity towards a range of wild-type and MDR cell lines.

Cytotoxic drug resistance frequently affects a wide range of cytotoxic agents. It is commonly inherent, although it may also be acquired after initial response to cytotoxic drugs. Cell lines exposed *in vitro* to one cytotoxic drug may also become resistant to a range of structurally and functionally diverse agents. This multidrug resistant (MDR) phenotype is associated with the presence of a 170 kd membrane glycoprotein, the P-glycoprotein (Pgp) (Juliano *et al.*, 1976; Kartner *et al.*, 1983; Riordan *et al.*, 1985) which acts as an energy dependent efflux pump (Skovsgaard, 1978). Many compounds have been shown to inhibit the action of Pgp thereby partly or completely overcoming drug resistance. Such resistance modifiers include verapamil (Tsuruo *et al.*, 1981) and other calcium antagonists, phenothiazines (Ford *et al.*, 1989) and cyclosporin A (Nooter *et al.*, 1989).

Many studies have attempted to reproduce the resistance modifying action of these drugs in a clinical setting. Unfortunately many cause unacceptable toxicity at levels below those needed to modulate resistance and have therefore not been useful clinically (Ozols *et al.*, 1987). Some encouraging results have however been reported in lymphoid malignancies. Verapamil and cyclosporin A appear to be able to reverse clinical drug resistance in myeloma and acute myelocytic leukaemia respectively (Dalton *et al.*, 1989; Nooter *et al.*, 1989).

The anti-oestrogen tamoxifen is one of the compounds that can modify multidrug resistance (Chatterjee *et al.*, 1990*b*). It is widely used in the treatment of breast cancer at doses of 20-40 mg day$^{-1}$ and at such doses produces serum levels around 0.5 μM (Adam *et al.*, 1980; Fabian *et al.*, 1980; Lien *et al.*, 1989). Doses up to 100 mg day$^{-1}$ have however been used with little toxicity, except for retinal toxicity after continuous treatment for periods of longer than 12 months (Kaiser-Kupfer *et al.*, 1978). High doses of tamoxifen may thus have the potential to produce serum levels sufficient to modulate multidrug resistance *in vitro* (3-10 μM) (Chatterjee *et al.*, 1990*b*; Foster *et al.*, 1988; Ramu *et al.*, 1984) making it an interesting compound to assess as a modifier of clinical drug resistance.

Etoposide (VP16-213, Vepesid) is an epipodophyllotoxin derived cytotoxic drug which interferes with the action of the nuclear enzyme topoisomerase II producing lethal single and double stranded DNA breaks (Chen *et al.*, 1984). It has activity in a number of solid tumours and resistance to it is part of the typical MDR phenotype (Endicott *et al.*, 1989). Etoposide is active orally and has manageable toxicity making it an attractive agent to combine with tamoxifen to produce an out-patient regimen.

We have therefore untaken a clinical study of oral etoposide in combination with intermittent, high-dose tamoxifen. This study has the dual aims of determining the maximum serum levels of tamoxifen and its metabolites that could be achieved and of assessing the toxicity and feasibility of the drug combination. The study therefore included patients with a range of relapsed and chemoresistant tumours. In conjunction with this we have assessed the *in vitro* effects of tamoxifen on etoposide cytotoxicity towards a panel of MDR + ve and MDR − ve cell lines and have compared this to the effects of the known MDR modulator verapamil.

### Materials and methods

### *Patient group*

Patients selected had histologically proven malignancy which had relapsed after or was resistant to standard therapy. Patients had ECOG performance scores of 2 or better, had normal renal and hepatic function and had normal peripheral blood counts. No patient had received systemic therapy in the 4 weeks prior to starting study treatment. The treatment protocol was approved by the local ethical committee and all patients gave informed consent prior to starting treatment.

### *Study protocol*

Treatment comprised high-dose oral tamoxifen combined with oral etoposide. Tamoxifen was given on days 1 to 6, etoposide on days 4 to 6 and treatment was repeated each 3 weeks. We have previously used tamoxifen at a dose of 320 mg day$^{-1}$ for 6 days (Millward *et al.*, 1992) and in this study the first nine patients received 480 mg day$^{-1}$. As no dose-limiting toxicity was noted the subsequent 16 patients received 720 mg day$^{-1}$ (12 20 mg tablets, three times day$^{-1}$).

Correspondence: N.S. Stuart.
Received 13 March 1992; and in revised form 8 July 1992.

No formal compliance check was conducted but patients were asked specifically whether they had difficulties taking the correct number of tablets. Oral etoposide was given at a dose of 300 mg $day^{-1}$ (150 mg two times $day^{-1}$) for the first course. Patients who remained myelosuppressed on day 21 had the dose of etoposide reduced to 200 mg $day^{-1}$ for subsequent courses. In those who had no significant myelosuppression the dose of etoposide was increased to 400 mg $day^{-1}$. Blood was taken for assessment of serum levels of tamoxifen and its metabolites on the morning of the fourth day of treatment between 1 and 4 h after the last dose and immediately prior to starting etoposide. A blood sample was also drawn on day 21 of the first course of treatment, i.e. after 15 days without tamoxifen and immediately before starting course 2. Blood samples were centrifuged and the serum removed and stored at −20°C prior to measurement.

*Determination of levels of tamoxifen and its metabolites*

Levels of tamoxifen and its metabolites were measured using high-performance liquid chromatography. The methods used have been published previously (Lien *et al.*, 1989; 1987). Briefly, tamoxifen and its metabolites were determined in the acetonitrile extract from serum and were separated by reverse-phase, low-dispersion, liquid chromatography. The drugs were detected by being converted to fluorophors by subjecting the effluent of the column to ultra-violet light while passing through a quartz tube. The identity of the analyses was confirmed by liquid chromatography/mass spectrometry using an on-line mass spectrometer connected to the analytical column (Lien *et al.*, 1988). Serum levels in different patient populations were compared using the Mann-Whitney U-test.

*Cells and culture conditions*

Three pairs of cell lines were assessed. Each comprised a wild-type parental cell line and a P-glycoprotein expressing daughter line. The wild-type MCF-7 cell line was derived from a human breast cancer (Soule *et al.*, 1973) while the CHO-K1 cell line was derived from Chinese hamster ovaries (CHO). The doxorubicin hydrochloride (dox)-resistant daughter lines of each (MCF-7 $adr^r$ and CHO-K1 $adr^r$ respectively) were developed by stepwise exposure to increasing dox concentrations (Batist *et al.*, 1986; Chatterjee *et al.*, 1990*a*). Both express Pgp and have a typical MDR phenotype. The S1 cell line (also known as SW-1573) was isolated from a human squamous cell lung cancer by Dr Leibovitz (Scott and White Clinic, Temple, TX). Its resistant offspring (S1/1.1) was produced by F. Baas (Neurozintuigenlab K2-214, 1105 AZ Amsterdam) by transfecting the S1 cell line with an expression vector (PFRCMV mdr1) bearing a full-length cDNA of the human *mdr*1 gene isolated from human liver using previously described methods (Lincke *et al.*, 1990). *mdr*1 expression in the S1/1.1 cell line is at least 100 fold higher than in the S1 line (F. Baas, personal communication) while *mdr*1 mRNA levels are about 5-fold higher than in the highly resistant 1R500 cell line produced from SW1573 by stepwise exposure to dox (Baas *et al.*, 1990).

Cell lines were grown at 37°C under 5% $CO_2$ in either RPMI 1640 (human cells) or Ham's F12 (CHO cells) medium, each supplemented with 10% foetal bovine serum and 2 mM glutamine. All cell lines were regularly subcultured to maintain them in exponential growth phase and were regularly tested to ensure they were free of mycoplasma infection.

*Drugs*

Etoposide (vepesid, VP16-213) formulated for intravenous, clinical use (Bristol-Myers Oncology, Slough, UK) was used in all experiments. Stock solutions as supplied by the manufacturers (20 mg $ml^{-1}$, 34 mM) was diluted in PBS to final concentration. Tamoxifen (supplied by ICI Pharmaceuticals PLC, Macclesfield, UK) and verapamil (Sigma Chemicals, Poole) were prepared as stock solutions at 50 mM in absolute ethanol and were stored at 4°C. Dilution to final concentration were made in PBS.

*Drug sensitivity assay*

The semi-automated, colorimetric, MTT assay was used to assess drug sensitivity (Carmichael *et al.*, 1987). Cells in exponential growth-phase were harvested by trypsinisation and seeded into 96-well plates in 180 µl fresh medium. The number of cells seeded varied from 2,000 to 8,000, according to the cell line, and was such that the control well was non-confluent at the end of the 4 day experiment. Drugs were added in a volume of 10 µl (PBS alone in control wells) to give a final volume of 200 µl. Cells were incubated for 4 days at 37°C under 5% $CO_2$. MTT was then added (50 µl of 2 mg $ml^{-1}$ = 0.1 mg), the cells incubated for a further 4 h and the medium removed by inverting the plate. The formazan crystals were then dissolved in 100 µl DMSO plus 25 µl glycine buffer pH 10.5 (Plumb *et al.*, 1989). Optical density at 540 nm was read on a Titertek Multiscan ELISA plate reader. Data was transferred to a Macintosh microcomputer and analysed using DeltaSoft software (BioMetallics Inc) which calculated $IC_{50}$ values. $IC_{50}$ was defined as the concentration of drug which reduced optical density to 50% of the control (non-drug) value.

The effect of tamoxifen on etoposide cytotoxicity was assessed at 1.5, 7.5 and 15 µM, the effect of verapamil was assessed at 6.6 µM. Maximum non-toxic concentration (MNTC) of tamoxifen was defined as the highest level that showed less than 10% growth inhibition when used alone. The extent of modulation of drug sensitivity was calculated by dividing the $IC_{50}$ for etoposide alone by that for etoposide in the presence of modifer. Enhancement values above 1.0 indicate enhancement of cytotoxicity while values below 1.0 indicate inhibition. Experiments were repeated at least three times and the values obtained under different conditions compared using the paired *t*-test.

### Results

*Clinical results*

Twenty-six patients were studied and their characteristics are listed in Table I. Median age was 50, median performance score 1. A range of histological tumour types were included with the dominant types being renal cell carcinoma ($n = 6$), gastrointestinal adenocarcinoma ($n = 4$), adenocarcinoma of unknown primary site ($n = 4$) and breast carcinoma ($n = 3$). Nine patients had received previous chemotherapy.

*Response* Two patients showed a partial response. One had intermediate grade lymphoma which had previously responded to combination chemotherapy including dox, cyclophosphamide and vincristine while the other had previously untreated adenocarcinoma of unknown primary site. Three patients had static disease for 24, 28 and 39 weeks, 16 patients showed no response and five patients died within two courses of treatment.

*Toxicity* The toxicity of the regimen is summarised in Table II. Nausea or vomiting occurred in four of 27 (15%) courses assessed at 480 mg $day^{-1}$ tamoxifen and in 10 of 30 (33%) courses assessed at 720 mg $day^{-1}$. Significant nausea was most commonly associated with the tamoxifen treatment and generally commenced before the etoposide therapy. A number of patients experienced subjective neurological symptoms. These were ill defined and were described as 'dizziness', unsteadiness of gait or marked malaise. These symptoms did not match standard WHO toxicity categories and were graded as mild (minimal symptoms), moderate (significant symptoms not requiring action), severe (significant symptoms requiring intervention) or very severe (unable to continue treatment). No objective neurological signs were noted. These

**Table I** Characteristics of patients included in clinical study

| | | |
|---|---|---|
| Number of patients entered on study | 26 | |
| Male: Female | 12:14 | |
| Median age (range) | 50 (36–77) | |
| Median ECOG score at start of treatment | 1 (0–2) | |
| Histological type | | Number with prior chemotherapy |
| Renal cell adenocarcinoma | 6 | 0 |
| Gastrointestinal adenocarcinoma | 4 | 0 |
| Adenocarcinoma of unknown primary site | 4 | 0 |
| Breast carcinoma | 3 | 3 |
| Soft tissue sarcoma | 2 | 2 |
| Bronchial carcinoma | 2 | 1 |
| Cervical carcinoma | 2 | 1 |
| Bladder, tonsil (squamous), high-grade lymphoma | 1 each | 2 |
| Tamoxifen dose received with first course | [a]160 mg = 1<br>480 mg = 9<br>720 mg = 16 | |

[a]160 mg day$^{-1}$ tamoxifen given to one patient with brain metastases.

**Table II** Toxicity of treatment

| | *Frequency (number of courses) and severity of nausea and vomiting* | | | | |
|---|---|---|---|---|---|
| *Tamoxifen dose* | *Grade 0* | *Grade 1/2* | *Grade 3/4* | *Not known* | *Total* |
| 480 mg day$^{-1}$ | 23 | 3 | 1 | 3 | 30 |
| 720 mg day$^{-1}$ | 20 | 6 | 4 | 5 | 35 |

| | *Number of patients experiencing neurological symptoms and grade* | | | |
|---|---|---|---|---|
| *Tamoxifen dose* | *None* | *Mild/moderate* | *Severe/v. severe* | *Total* |
| 480 mg day$^{-1}$ | 7 | 3 (30%) | 0 | 10 |
| 720 mg day$^{-1}$ | 11 | 4 (25%) | 1 (6%) | 16 |

symptoms occurred in three of ten patients while receiving 480 mg day$^{-1}$ tamoxifen and in five of 16 while receiving 720 mg day$^{-1}$. Seven hundred and twenty mg day$^{-1}$ was considered the maximum tolerated dose of tamoxifen given by this schedule.

Twelve of 35 cycles of treatment at 300 mg day$^{-1}$ of etoposide were associated with leucopenia (WHO grade three or four in four courses). No significant thrombocytopenia was recorded. Six patients had their etoposide dose reduced from 300 to 200 mg day$^{-1}$ because of myelosuppression while in three the dose was increased from 300 to 400 mg day$^{-1}$. No patients had to stop treatment because of toxicity.

*Pharmacokinetics*

The level of tamoxifen and two of its metabolites on the morning of day 4 of the first three courses of treatment at 480 and 720 mg day$^{-1}$ are shown in Figure 1. Tamoxifen levels averaged 3.5 μM after the first 3 days of treatment at 480 mg day$^{-1}$ and 3.2 μM after 3 days of treatment at 720 mg day$^{-1}$ (not significantly different). Levels of N-desmethyltamoxifen were lower than those of tamoxifen (2.1 μM and 2.2 μM after the first 3 days of treatment at 480 and 720 mg day$^{-1}$ respectively), while didesmethyltamoxifen were about 5–10% of tamoxifen levels during the first course (0.15 μM and 0.14 μM after 480 and 720 mg day$^{-1}$ respectively). After completing 6 days treatment with 720 mg day$^{-1}$ levels of tamoxifen fell to below 1 μM by day 21 but N-desmethyltamoxifen levels remained static (2.1 μM on day 21) while didesmethyltamoxifen levels rose, reaching 0.5 μM.

Levels of tamoxifen and its metabolites on day 3 of the first three courses were not significantly different in patients receiving 720 mg day$^{-1}$ compared to those receiving 480 mg day$^{-1}$. Similarly levels of tamoxifen on day 3 of course 1

**Figure 1** Serum levels (μM ± s.e. where $n > 2$) of tamoxifen (□), N-desmethyltamoxifen (◇) and didesmethyltamoxifen (△) on the fourth day of the first three 6 day courses at two tamoxifen dose levels. *,† = levels significantly different, $P < 0.01$.

were not statistically different to those on day 3 of subsequent courses. N-desmethyltamoxifen levels were however, higher in course 2 than in course 1. Similar levels were achieved after 480 mg day$^{-1}$ (2.1 μM during course 1 *vs* 3.2 μM during course 2) to those after 720 mg day$^{-1}$ (2.2 μM *vs* 3.36 μM) but only in the latter case was this difference significant ($P<0.01$). Didesmethyltamoxifen levels were also significantly higher during course 2 that during course 1 after both 480 mg day$^{-1}$ (0.15 μM rising to 0.45 μM, $P<0.001$) and 720 mg day$^{-1}$ (0.14 μM rising to 0.48 μM, $P<0.01$). Didesmethyltamoxifen levels however, remained below 1.0 μM.

### In vitro *effects*

Table III and IV show the effects of tamoxifen and verapamil respectively, on the cytotoxicity of etoposide towards the cell lines used. In comparison to the MCF-7 wild type cell line the MCF-7 adr$^r$ cell line showed 60-fold resistance to etoposide while the CHO-K1 adr$^r$ line was 5-fold more resistant than its parental line. The S1/1.1 cell line, which has been transfected with the *mdr*1 gene, was however 2-fold more sensitive than the parental line ($P<0.01$). Tamoxifen produced a minor degree of enhancement of etoposide cytotoxicity in the MCF-7 parental cell line reducing the $IC_{50}$ from 0.95 μM to 0.77 μM ($P<0.01$) but had the opposite effect in the MCF-7 adr$^r$ line where $IC_{50}$ increased from 60 μM to 84 μM ($P<0.005$). Tamoxifen had no significant effect on the cytotoxicity of etoposide towards the S1, S1/1.1, CHO-K1 or CHO-K1 adr$^r$ cell lines. Verapamil at 6.6 μM had a similar effect to tamoxifen on the cell lines producing enhancement of etoposide cytotoxicity in the MCF-7 wt cell line ($P<0.03$), minor inhibition in the MCF-7 adr$_r$ cell line and no significant effect on the CHO-K1 and S1 cell lines.

### Discussion

We report here both clinical and *in vitro* studies of the effects of tamoxifen on etoposide cytotoxicity and also the serum levels of tamoxifen and its metabolites that can be achieved with intermittent high-dose tamoxifen. The clinical study assessed a range of solid tumours and showed two responses among 26 patients (in relapsed lymphoma and in untreated adenocarcinoma). As this was a phase I study designed to assess the feasibility of administering the combination of drugs it is not possible to determine the contribution of the tamoxifen to these responses as both tumour types can respond to etoposide alone. Treatment toxicity was generally manageable and was less marked than that reported with 480 mg day$^{-1}$ continuously (Stuart *et al.*, 1991). Nevertheless, we consider that 720 mg day$^{-1}$ is the maximum tolerated dose for tamoxifen given by this schedule. Thirty-three percent of courses at 720 mg day$^{-1}$ were associated with nausea or vomiting although no patient stopped treatment because of this. Neurological toxicity occurred in a number of patients taking both 480 and 720 mg day$^{-1}$ and comprised the same subjective symptoms noted by us in patients taking 480 mg day$^{-1}$ continuously (Stuart *et al.*, 1991) as well as by others (Trump *et al.*, 1991). The mechanism of such toxicity is not known but Lien *et al.* have recently reported levels of tamoxifen in brain metastases and in normal brain tissue in four patients taking standard dose tamoxifen (Lien *et al.*, 1991). They showed that brain levels are some 46-fold higher than serum levels. Although the distribution of tamoxifen at high doses may be different from the standard doses assessed by Lien *et al.*, it is likely that brain levels are considerably higher than the serum levels reported in the present study. It is also possible that tamoxifen alters the tissue distribution of etoposide rendering it more neurotoxic. The possibility that resistance modifiers may exacerbate the normal tissue toxicity of chemotherapy has been raised by one non-randomised study (Berd *et al.*, 1991). The extent of myelosuppression observed in this study was similar to that expected.

Several studies have described the pharmacokinetics of standard dose tamoxifen (20–40 mg day$^{-1}$) and the levels of its metabolites that result (Adam *et al.*, 1980; Fabian *et al.*, 1980; Lien *et al.*, 1989). These studies show steady state serum levels of tamoxifen of around 0.25–1.0 μM after 4 weeks. NDMTx accumulated more slowly to levels some 50% higher after 8 weeks. diDMTx levels are generally about 60–85% lower than those of the parent compound. These ratios are similar to those in the present study but the levels produced by high-dose therapy are lower than expected, indicating non-linear pharmacokinetics. The fact that serum levels were not significantly higher after 720 mg day$^{-1}$ than

**Table III** Cytotoxicity of etoposide and tamoxifen towards each cell line ($IC_{50}$), maximum non-toxic concentration of tamoxifen (MNTC), $IC_{50}$ in the presence of MNTC, degree and significance of enhancement

| *Cell line* | *$IC_{50}$ etoposide (μM ± s.e)* | *$IC_{50}$ tamoxifen (μM)* | *MNTC tamoxifen (μM)* | *$IC_{50}$ etoposide at MNTC tamoxifen (μM ± s.e.)* | *Enhancement* | *P-value* |
|---|---|---|---|---|---|---|
| MCF-7 wt | 0.95 ± 0.12 | 8.3 | 0.15 | 0.77 ± 0.09 | 1.2 | $P<0.01$ |
| MCF-7 adr$^r$ | 60 ± 6 | 20 | 7.5 | 89. ± 7 | 0.7 | $P<0.005$ |
| CHO-K1 | 1.2 ± 0.45 | 10 | 1.5 | 1.2 ± 0.46 | 1.0 | n.s. |
| CHO-K1 adr$^r$ | 5.2 ± 1.68 | 7.8 | 1.5 | 6.1 ± 1.63 | 0.85 | n.s. |
| S1 | 2.8 ± 0.51 | 12 | 7.5 | 3.1 ± 0.97 | 0.9 | n.s. |
| S1/1.1 | 1.7 ± 0.39 | 12 | 7.5 | 1.96 ± 0.56 | 0.9 | n.s. |

Values represent mean value of at least three experiments.

**Table IV** Cytotoxicity of etoposide towards each cell lines ($IC_{50}$), $IC_{50}$ in the presence of 6.6 μM verapamil and degree and significance of enhancement

| *Cell line* | *$IC_{50}$ etoposide (μM ± s.e.)* | *$IC_{50}$ etoposide at 6.6 μM verapamil (μM ± s.e.)* | *Enhancement* | *P-value* |
|---|---|---|---|---|
| MCF-7 wt | 1.02 ± 0.21 | 0.54 ± 0.11 | 1.9 | <0.03 |
| MCF-7 adr$^r$ | 66 ± 6 | 91 ± 11 | 0.73 | n.s. |
| CHO-K1 | 1.2 ± 0.57 | 0.76 ± 0.36 | 1.6 | n.s. |
| CHO-K1 adr$^r$ | 5.6 ± 1.97 | 4.4 ± 4.38 | 1.3 | n.s. |
| S1 | 3.6 ± 0.79 | 3.0 ± 0.76 | 1.2 | n.s. |
| S1/1.1 | 1.5 ± 0.32 | 1.6 ± 0.37 | 0.9 | n.s. |

Values represent mean value of at least three experiments.

after 480 mg $day^{-1}$ supports this. Non-linear pharmacokinetics may be due to lower bioavailability of high oral doses or to saturation of portal vein plasma proteins leading to increased hepatic, pre-systemic metabolism. However, patients on the higher dose were asked to take 36 tablets each day and it is possible that compliance was incomplete. We cannot exclude the possibility that the apparent non-linear pharmacokinetics were due, in part, to failure of patients to take the full dose. Tamoxifen also has a very high volume of distribution (Lien *et al.*, 1989) and higher doses may have produced increased drug influx into the tissues. Blood sampling took place 1 to 4 h after treatment but did not necessarily coincide with peak levels. The influx into tissues may have been promoted by peak serum levels higher than those recorded. The greater toxicity of higher doses suggests that they produce higher levels in some tissues even though serum levels were similar.

The slower fall in levels of NDMTx and diDMTx while not taking tamoxifen, and the accumulation with subsequent courses is consistent with previous reports of the longer half-life of these compounds (Adam, 1981; Lien *et al.*, 1987). The finding of non-linear pharmacokinetics is of clinical relevance in that it limits the ability to achieve high serum levels. A dose of 720 mg $day^{-1}$ for 3 days produces levels no higher than 480 mg $day^{-1}$ (about 3 μM) while continuing 480 mg $day^{-1}$ indefinitely raises levels only marginally to around 4 μM (Stuart *et al.*, 1991). Trump *et al.* have reported somewhat higher serum levels of both tamoxifen (6.1 μM) and NDMTx (6.6 μM) after 9 days on 260 mg of tamoxifen twice a day (Trump *et al.*, 1991). Full pharmacokinetics were though, not reported, so it is difficult to relate these findings to the present study. Trump *et al.* used more prolonged treatment and reported serum levels on day 9 to 13. We have previously shown that when taking 160 mg three times a day serum levels of tamoxifen plateau at around 4 μM after 10 days while levels of NDMTx reach 7 μM after 21 days (Stuart *et al.*, 1991). Levels would therefore be expected to be higher on day 9 to 13 than on day 4, as in the present study. Timing of serum samples in relation to peak levels may be important as may differences in methodology – Trump *et al.* give no details of their methodology. Their report is however, consistent with the results of the present study and supports the non-linear pharmacokinetics of tamoxifen.

The *in vitro* studies reported here show that neither tamoxifen nor verapamil has a major effect on etoposide cytotoxicity in the cell lines assessed. Tamoxifen produced a statistically significant enhancement of etoposide cytotoxicity in the MCF-7 wt cell line and had the opposite effect in the MCF-7 $adr^4$ line while verapamil produced similar effects. These changes were, however, modest in extent (maximum enhancement 1.9-fold) and too small to be of any clinical significance. Previous reports have shown that tamoxifen can reverse drug resistance in a number of *in vitro* models but these have mainly assessed adriamycin cytotoxicity. Our group has reported that tamoxifen enhances dox cytotoxicity in the MCF-7 $adr^r$ cell line and has similar effects on vinblastine cytotoxicity in the S1/1.1 and CHO-K1 $adr^r$ (Kirk *et al.*, 1991 #117; Kirk, 1992; in press). Ramu *et al.* initially assessed the effects of a range of triparanol compounds, including tamoxifen, on adriamycin cytotoxicity towards the P388 murine leukaemia line and its adriamycin resistant subline (P388/ADR) (Ramu *et al.*, 1984). They showed that 3 μM tamoxifen partially reversed the resistance of the P388/ADR line. Foster *et al.* assessed both adriamycin and vinblastine cytotoxicity in the MCF-7 $adr^r$ cell line used in the present study and also showed partial reversal in the presence of 10 μM tamoxifen (Foster *et al.*, 1988). The effect of tamoxifen on dox cytotoxicity towards the CHO-K1 and CHO-K1 $adr^r$ cell lines has also been assessed by Chatterjee and Harris who have shown enhancement of cytotoxicity in both the parental (4.8-fold) and dox resistant lines (16-fold) (Chatterjee *et al.*, 1990*b*). Other reports confirm the resistance modifying effects of tamoxifen (Berman *et al.*, 1991) but none have assessed its effects on etoposide cytotoxicity.

The mechanism whereby tamoxifen modified dox and vinblastine cytotoxicity is not known. Tamoxifen does not appear to bind to Pgp (Kessel, 1986) but it does inhibit both calmodulin (Tsuruo *et al.*, 1983) and protein kinase C (Horgan *et al.*, 1986). Both enzymes have been suggested as possible mechanisms by which drug resistance could be modified. PKC phosphorylates and activates Pgp (Chambers *et al.*, 1990) while inhibition of PKC downregulates Pgp function and enhances drug cytotoxicity (Ma *et al.*, 1991). Other mechanisms independent of PgP, by which cytotoxicity could be modulated, have also been suggested (Baas *et al.*, 1990; Hindenburg *et al.*, 1987).

Many other drugs have been reported to modify multidrug resistance and verapamil is perhaps the most studied of these (Ford *et al.*, 1990). These studies have concentrated on enhancement of vinblastine and dox cytotoxicity but some have assessed multidrug resistance in more detail. Merry *et al.* have used an *in vivo* model and have developed a drug resistant variant of the Ridgway osteogenic sarcoma (Merry *et al.*, 1991). This showed moderate resistant to actinomycin-D (1.5 fold), vincristine (3.5-fold) and etoposide (5-fold). In this case verapamil was equally effective in reversing the resistance to each agent. Other work using Chinese hamster lung (DC3F/ADX) and breast cancer (MCF-7 $adr^r$) cell lines has shown that, while verapamil effectively reverses resistance to vincristine in multidrug resistant mutants (11–125 fold enhancement), it has only modest effects on etoposide resistance (3-4 fold) (Politi *et al.*, 1990). Politi *et al.* also showed that verapamil had little effect on intracellular etoposide accumulation and that etoposide had lower affinity for Pgp than vincristine. Beck *et al.* have also assessed the ability of verapamil to overcome multidrug resistance in a human leukaemic cell line (CEM/VBL100) and have confirmed a modifying effect on the cytotoxicity of vinca alkaloids (Beck *et al.*, 1986). In this case other drugs (including doxorubicin and etoposide) were unaffected. It therefore appears that reversal of multidrug resistance is not an 'all or none' effect and various elements of the multidrug resistant phenotype can be differentially affected.

Although Pgp expression alone is sufficient for the development of the MDR phenotype (Lincke *et al.*, 1990), it is known that many other mechanisms exist. The observation that resistance modifiers known to act on Pgp only partially reverse drug resistance in Pgp expressing cell lines (Politi *et al.*, 1989) points to the presence of additional resistance mechanisms in these cell lines. Where Pgp plays only a minor role, altering its function may shcw little effect on overall levels of resistance. Lincke *et al.* report that transfection of the *mdr*1 gene into chemosensitive human BRO melanoma cells produces a typical MDR phenotype which can be partially reversed with verapamil (Lincke *et al.*, 1990). The S1 parental cell line used in the present study was transfected with the same gene but has much higher inherent resitance than the parental BRO cell line ($IC_{50}$ for etoposide; S1 = 3 μM, BRO = 0.016 μM). In the S1/1.1 cell line other resistance mechanisms are likely to be present explaining the lack of effect of verapamil. Certain of these mechanisms may themselves be potential targets for modification. Changes in topoisomerase II, in site of activity of etoposide, may account for certain patterns of drug resistance. Low levels of topoisomerase expression have been related to etoposide (Kim *et al.*, 1992) as well as to drugs with other sites of action (Giaccone *et al.*, 1992). Phosphorylation of topoisomerase II can also enhance resistance to etoposide (Devore *et al.*, 1992) and would be a potential route of modulation of drug resistance.

The clinical study reported here shows that tamoxifen at a dose of either 480 mg $day^{-1}$ or 720 mg $day^{-1}$ can achieve, by the 4th day, serum levels of tamoxifen sufficient to modulate drug resistance in other *in vitro* models. When given for up to 6 days such doses produce acceptable toxicity. The value of tamoxifen as an *in vivo* modulator of drug resistance therefore deserves further study. For clinical studies we recommend intermittent high-doses of tamoxifen (480 mg $day^{-1}$) combined with cytotoxic drugs whose toxicity is significantly modulated *in vitro*. Compounds related to tamoxifen such as toremifene also need to be assessed in this context. Toremifene can be administered at higher doses than tamoxifen (Kohler et al., 1990; Robinson et al., 1990) and higher serum levels can be achieved (DeGregorio et al., 1989; Kaye, 1990). It is also an effective enhancer of drug toxicity in MDR-positive cell lines (Kirk, 1992; in press).

The *in vitro* data show that, although etoposide resistance is an accepted part of the MDR phenotype, this resistance is not affected by tamoxifen or verapamil which are effective modulators of the cytotoxicity of other drugs that are part of the MDR phenotype. Other studies however, show that novel modulators such as cyclosporin analogues may have a much greater effect on etoposide cytotoxicity (Gaveriaux et al., 1991). Future clinical studies using modifiers of multidrug resistance need to select cytotoxic drugs that have been shown to be modulated *in vitro* by the modifier being assessed. The combination of etoposide and tamoxifen is a poor candidate for such studies.

### References

ADAM, H.K. (1981). A review of the pharmacokinetics and metabolism of 'Nolvadex' (Tamoxifen). In: *Non-steroidal Antiestrogens*. Sutherland R.L. & Jordan, V.C. (eds). Academic Press: Sidney.

ADAM, H.K., PATTERSON, J.S. & KEMP, J.V. (1980). Studies on the metabolism and pharmacokinetics of tamoxifen in normal volunteers. *Cancer Treat. Rep.*, **64**, 761–764.

BAAS, F., JONGSMA, A.P.M., BROXTERMAN, H.J., ARCECI, R.J., HOUSMAN, D., SCHEFFER, G.L., RIETHORST, A., VAN GROENIGEN, M., NIEUWINT, A.W.M. & JOENJ, H. (1990). Non-P-glycoprotein mediated mechanism for multidrug resistance precedes P-glycoprotein expression during *in vitro* selection for doxorubicin resistance in a human lung cancer cell line. *Cancer Res.*, **50**, 5392–5398.

BATIST, G., TULPULE, A., SINHA, B.K., KATKI, A.G., MYERS, C.E. & COWAN, K.H. (1986). Overexpression of a novel anionic glutathione transferase in multidrug resistant human breast cancer cells. *J. Biol. Chem.*, **261**, 15544–15549.

BECK, W.T., CIRTAIN, M.C., LOOK, A.T. & ASHMUN, R.A. (1986). Reversal of vinca alkaloid resistance but not multiple drug resistance in human leukemic cells by verapamil. *Cancer Res.*, **46**, 778–784.

BERD, D., MCLAUGHLIN, C.J., HART, E., WIEBE, V.J., MASTRANGELO, R., BELLET, R.E. & DEGREGORIO, M.W. (1991). Short course high-dose tamoxifen (TAM) with cytotoxic chemotherapy for metastatic melanoma. *Proc. Am. Soc. Clin. Oncol.*, **10**, 291.

BERMAN, E., ADAMS, M., DUIGOU-OSTERNDORF, R., GODFREY, L., CLARKSON, B. & ANDREEFF, M. (1991). Effect of tamoxifen on cell lines displaying the multidrug resistant phenotype. *Blood*, **77**, 818–825.

CARMICHAEL, J., DEGRAFF, W.G., GAZDAR, A.F., MINNA, J.D. & MITCHELL, J.B. (1987). Evaluation of a tetrazolium based semiautomated colorimetric assay: assessment of chemosensitivity testing. *Cancer Res.*, **47**, 936–942.

CHAMBERS, T.C., MCAVOY, E.M., JACOBS, J.W. & EILON, G. (1990). Protein kinase C phosphorylates P-glycoprotein in multidrug resistant human KB carcinoma cells. *J. Biol. Chem.*, **265**, 7679–7686.

CHATTERJEE, M. & HARRIS, A.L. (1990*a*). Enhancement of adriamycin cytotoxicity in a multidrug resitant Chinese hamster ovary (CHO) subline, CHO-Adr$^r$, by Toremifene and its modulation by alpha$_1$ acid glycoprotein. *Eur. J. Cancer*, **26**, 432–436.

CHATTERJEE, M. & HARRIS, A.L. (1990*b*). Reversal of acquired resistance to adriamycin in CHO cells by tamoxifen and 4-hydroxy tamoxifen: role of drug interaction with alpha 1 acid glycoprotein. *Br. J. Cancer*, **62**, 712–717.

CHEN, G.L., YANG, L., ROWE, T.C., HALLIGAN, B.D., TEWEY, K.M. & LIU, L.F. (1984). Nonintercalative antitumour drugs interfer with the breakage-reunion reaction of mammalian DNA topoisomerase II. *J. Biol. Chem.*, **259**, 13560–13566.

DALTON, W.S., GROGAN, T.M., MELTZER, P.S., SCHEPER, R.J., DURIE, B.G.M., TAYLOR, C.W., MILLER, T.P. & SALMON, S.E. (1989). Drug-resistance in multiple myeloma and non-Hodgkin's lymphoma: detection of P-glycoprotein and potential circumvention by addition of verapamil to chemotherapy. *J. Clin. Oncol.*, **7**, 415–424.

DEGREGORIO, M.W., FORD, J.M., BENZ, C.C. & WIEBE, V.J. (1989). Toremifene: pharmacologic and pharmacokinetic basis of reversing multidrug resistance. *J. Clin. Oncol.*, **7**, 1359–1364.

DEVORE, R.F., CORBETT, A.H. & OSHEROFF, N. (1992). Phosphorylation of topoisomerase II by casein kinase II and sensitivity to the antineoplastic drugs etoposide and 4'-(9-acridinylamino)-methanesulfon-m-anisidide. *Cancer Res.*, **52**, 2156–2161.

ENDICOTT, J.A. & LING, V. (1989). The biochemistry of P-glycoprotein-mediated multidrug resistance. *Annu. Rev. Biochem.*, **58**, 137–171.

FABIAN, C., STERNSON, L. & BARNETT, M. (1980). Clinical pharmacology of tamoxifen in patients with breast cancer: comparison of traditional and loading dose schedules. *Cancer Treat. Rep.*, **64**, 761–764.

FORD, J.M. & HAIT, W.N. (1990). Pharmacology of drugs that alter multidrug resistance in cancer. *Pharmacol. Rev.*, **42**, 155–199.

FORD, J.M., PROZIALECK, W.C. & HAIT, W.N. (1989). Structural features determining activity of phenothiazines and related drugs for inhibition of cell growth and reversal of multidrug resistance. *Mol. Pharmacol.*, **35**, 105–115.

FOSTER, B.J., GROTZINGER, K.R., MCKOY, W.M., RUBINSTEIN, L.V. & HAMILTON, T.C. (1988). Modulation of induced resistance to adriamycin in two human breast cancer cell lines with tamoxifen or perhexiline maleate. *Cancer Chemother. Pharmacol.*, **22**, 147–152.

GAVERIAUX, C., BOESCH, D., JACHEZ B., BOLLINGER, P., PAYNE, T. & LOOR, F. (1991). SDZ-PSC-833, a non-immunosuppressive cyclosporin analog, is a very potent multidrug-resistance modifier. *J. Cell. Pharmacol.*, **2**, 225–234.

GIACCONE, G., GAZDAR, A.F., BECK, H., ZUNINO, F. & CAPRANICO, G. (1992). Multidrug sensitivity phenotype of human lung-cancer cells associated with topoisomerase II expression. *Cancer Res.*, **52**, 1666–1674.

HINDENBERG, A.A., BAKER, M.A., GLEYZER, E., STEWART, V.J., CASE, N. & TAUB, R.N. (1987). Effect of verapamil and other agents on the distribution of anthracyclines and on reversal of drug resistance. *Cancer Res.*, **47**, 1421–1425.

HORGAN, K., COOKE, E., HALLETT, M.B. & MANSEL, R.E. (1986). Inhibition of protein kinase C mediated signal transduction by tamoxifen. *Biochem. Pharm.*, **35**, 4463–4465.

JULIANO, R.L. & LING, V. (1976). A surface glycoprotein modulating drug permeability in Chinese hamster ovary cell mutants. *Biochim. Biophys. Acta.*, **455**, 152–162.

KAISER-KUPFER, M.I. & LIPPMAN, M. (1978). Tamoxifen retinopathy. *Cancer Treat. Rep.*, **62**, 321–325.

KARTNER, N., RIORDAN, J.R. & LING, V. (1983). Cell surface p-glycoprotein associated with multidrug resistance in mammalian cell lines. *Science*, **221**, 1285–1288.

KAYE, S.B. (1990). Reversal of multidrug resistance. *Cancer Treat. Rep.*, **17** (Supplement A), 37–43.

KESSEL, D. (1986). Interactions among membrane transport systems: anthracyclines, calcium antagonisms and anti-oestrogens. *Biochem. Pharm.*, **35**, 2825–2826.

KIM, R., HIRABAYASHI, N., NISHIYAMA, M., JINUSHI, K., TOGE, T. & OKADA, K. (1992). Factors contributing to adriamycin sensitivity in human xenograft tumors – the relationship between expression of the MDR1, GST-pi and topoisomerase II genes and tumor sensitivity to adriamycin. *Anticancer Res.*, **12**, 241–245.

KIRK, J., HOULBROOK, S., STUART, N.S., STRATFORD, I.J., HARRIS, A.L. & CARMICHAEL, J. (1991). Effects of anti-oestrogens on adriamycin cytotoxicity to breast and lung cancer cell lines. *Br. J. Cancer*, **63** (suppl XIII), 44.

KOHLER, P.C., HAMM, J.T., WIEBE, V.J., DEGREGORIO, M., SHEMANO, I. & TORNEY, D.C. (1990). Phase I study of the tolerance and pharmacokinetics of toremifene in patients with cancer. *Breast Cancer Res. Treat.*, **16**, S10–S26.

LIEN, E.A., SOLHEIM, E., KVINNSLAND, S. & UELAND, P.M. (1988). Identification of 4-hydroxy-N-desmethyltamoxifen as a metabolite of tamoxifen in human bile. *Cancer Res.*, **48**, 2304–2308.

LIEN, E.A., SOLHEIM, E., LEA, O.A., LUNDGREN, S., KVINNSLAND, S. & UELAND, P.M. (1989). Distribution of 4-hydroxy-N-desmethyltamoxifen and other tamoxifen metabolites in human biological fluids during tamoxifen treatment. *Cancer Res.*, **49**, 2175–2183.

LIEN, E.A., UELAND, P.M., SOLHEIM, E. & KVINNSLAND, S. (1987). Determination of tamoxifen and four metabolites in serum by low-dispersion liquid chromatography. *Clin. Chem.*, **33**, 1608–1614.

LIEN, E.A., WESTER, K., LONNING, P.E., SOLHEIM, E. & UELAND, R.M. (1991). Distribution of tamoxifen and metabolites into brain tissue and brain metastases in breast cancer patient. *Br. J. Cancer*, **63**, 641–645.

LINCKE, C.R., VAN DER BLIEK, A.M., SCHUURHUIS, G.J., VAN DER VELDE-KOERTS, T., SMIT, J.J.M. & BORST, P. (1990). Multidrug resistance phenotype of human BRO melanoma cells transfected with a wild-type human *mdr*1 complementary DNA. *Cancer Res.*, **50**, 1779–1785.

MA, L., MARQUARDT, D., TAKEMOTO, L. & CENTRE, M.S. (1991). Analysis of P-glycoprotein phosphorylation in HL60 cells isolated for resistance to vincristine. *J. Biol. Chem.*, **266**, 5593–5599.

MERRY, S., HAMILTON, T.G., FLANIGAN, P., FRESHNEY, R.I. & KAYE, S.B. (1991). Circumvention of pleiotropic drug resistance in subcutaneous tumous *in vivo* with verapamil and clomipramine. *Eur. J. Cancer*, **27**, 31–34.

MILLWARD, M.J., CANTWELL, B.M.J., LIEN, E., CARMICHAEL, J. & HARRIS, A.L. (1992). Intermittent high dose tamoxifen as a potential modifier of multi-drug resistance: a phase I/II study. *Eur. J. Cancer*, **28A**, 805–810.

NOOTER, K., OOSTRUM, R., JONKER, R., VAN DEKKEN, H., STOKDIJK, W. & VAN DEN ENGH, G. (1989). Effect of cyclosporin A on daunorubicin accumulation in multidrug-resistant P388 leukemia cells measured by realtime flow cytometry. *Cancer Chemother. Pharmacol.*, **23**, 296–300.

OZOLS, R.F., CUNNION, R.E., KLECKER, R.W., HAMILTON, T.C., OSTCHEGA, Y., PARRILLO, J.E. & YOUNG, R.C. (1987). Verapamil and adriamycin in the treatment of drug-resistant ovarian cancer patients. *J. Clin. Oncol.*, **5**, 641–647.

PLUMB, J.A., MILROY, R. & KAYE, S.B. (1989). Effects of the pH dependence of 3-(4,5-dimethylthiazol-2-yl)-2,5-diphenyl tetrazolium bromide-formazan absorption on chemosensitivity determined by a novel tetrazolium-based assay. *Cancer Res.*, **49**, 4435–4440.

POLITI, P.M., ARNOLD, S.T., FELSTED, R.L. & SINHA, B.K. (1990). P-glycoprotein-independent mechanism of resistance to VP-16 in multidrug-resistant tumour cell lines: pharmacokinetic and photoaffinity labeling studies. *Mol. Pharmacol.*, **37**, 790–796.

POLITI, P.M. & SINHA, B.K. (1989). Role of differential drug uptake, efflux, and binding of etoposide in sensitive and resistant human tumour cell lines: implications for the mechanisms of drug resistance. *Mol. Pharmacol.*, **35**, 271–278.

RAMU, A., GLAUBIGER, D. & FUKS, Z. (1984). Reversal of acquired resistance to doxorubicin in P388 murine leukaemia cells by tamoxifen and triparanol analogues. *Cancer Res.*, **44**, 4392–4395.

RIORDAN, J.R. & LING, V. (1985). Genetic and biochemical characterization of multidrug resistance. *Pharmacol. Therapy*, **28**, 51–75.

ROBINSON, S.P., PARKER, C.J. & JORDAN, V.C. (1990). Preclinical studies with toremifene as an antitumour agent. *Breast Cancer Res. Treat.*, **16**, S9–S18.

SKOVSGAARD, T. (1978). Mechanisms of resistance to daunorubicin in Ehrlich ascites tumour cells. *Cancer Res.*, **38**, 1785–1791.

SOULE, H.D., VAZQUEZ, J., LONG, A., ALBERT, S. & BRENNAN, M. (1973). A human cell line from a pleural effusion derived from a breast cancer. *J. Natl Cancer Inst.*, **41**, 1409–1416.

STUART, N.S.A., PHILIP, P., HARRIS, A.L., TONKIN, K., HOULBROOK, S., KIRK, J., LIEN, E., BENSON, M., LANE, D. & CARMICHAEL, J. (1991). Tamoxifen enhancement of etoposide cytotoxicity in lung cancer: a combined clinical and *in vitro* study (in press).

TRUMP, D.L., SMITH, D.C., SCHOLD, S.C., ROGERS, M.P., ELLIS, P.G., FINE, R.L., WINER, E.P., PANELLA, T.J., MOORE, J.O., HATHORN, J., GOCKERMAN, J. & JORDAN, V.C. (1991). High dose tamoxifen and five day continuous infusion of vinblastine: a phase I trial of an inhibitor of the MDR-1 phenotype. *Proc. Am. Soc. Clin. Oncol.*, **10**, 96.

TSURUO, T., IIDA, H., TSUKAGOSHI, S. & SAKURAI, Y. (1981). Overcoming of vincristine resistance in P388 leukemia *in vivo* and *in vitro* through enhanced cytotoxicity of vincristine and vinblastine by verapamil. *Cancer Res.*, **41**, 1967–1972.

TSURUO, T., IIDA, H., TSUKAGOSHI, S. & SAKURAI, Y. (1983). Potentiation of vincristine and adriamycin effects in human hemopoietic tumour cell lines by calcium antagonists and calmodulin inhibitors. *Cancer Res.*, **43**, 2267–2272.